# Additional Value of Ultrasound in Patients with Psoriatic Arthritis within Treatment Target

**DOI:** 10.3390/jcm13154567

**Published:** 2024-08-05

**Authors:** Mihaela Agache, Claudiu C. Popescu, Luminița Enache, Corina Mogoșan, Emilio Filippucci, Cătălin Codreanu

**Affiliations:** 1 Rheumatology Department, “Carol Davila” University of Medicine and Pharmacy, 020021 Bucharest, Romania; mihaela.agache@reumatologiedrstoia.ro (M.A.); luminita.enache@reumatologiedrstoia.ro (L.E.); corina.mogosan@reumatologiedrstoia.ro (C.M.); catalin.codreanu@reumatologiedrstoia.ro (C.C.); 2 “Ion Stoia” Clinical Center for Rheumatic Diseases, 020983 Bucharest, Romania; 3 Rheumatology Unit, Department of Clinical and Molecular Sciences, “Carlo Urbani” Hospital, Polytechnic University of Marche, 60035 Ancona, Italy; emilio.filippucci@yahoo.it

**Keywords:** psoriatic arthritis, ultrasound, enthesitis

## Abstract

**Background:** In psoriatic arthritis (PsA), musculoskeletal ultrasound is a complementary tool to physical examination, useful even in patients in remission to detect subclinical activity. **Objectives:** The objective of the study was to assess the ultrasound prevalence of active enthesitis and synovitis in patients who reached the therapeutic target. **Methods:** This cross-sectional study included patients with at least 6 months of therapy with a targeted synthetic or biological disease-modifying antirheumatic drug who were in treatment target (i.e., DAPSA < 14). Patients underwent bilateral clinical and ultrasound examination of the elbow lateral epicondyle, quadriceps insertion, distal patellar tendon insertion, and Achilles enthesis for assessing enthesitis, and hand and foot joints for assessing synovitis. Enthesitis and synovitis were considered active if the power Doppler signal showed at least a score of one. **Results:** The study included 51 PsA patients, women (52.9%), with an average age of 55 years. Although the patients were within the DAPSA treatment target, 21.6% had at least one painful enthesis at clinical examination, 19.6% had ultrasound evidence of at least one active enthesitis and 15.7% had ultrasound signs of at least one active synovitis. **Conclusions:** Among PsA patients thought to be within the therapeutic target, ultrasound detected a non-negligible percentage of active enthesitis and synovitis.

## 1. Introduction

Psoriatic arthritis (PsA) is a chronic and complex inflammatory joint disease with a high variability regarding its clinical phenotype, with six major domains defined by the GRAPPA-OMERACT PsA working group [1]. The diagnosis, the assessment of the disease’s domains, as well as the therapeutic approach (either initiation or monitoring) still pose a challenge for the rheumatologist. The treatment’s target is achieving remission or low disease activity (LDA) in order to avoid structural progression [2]. Conventional synthetic (csDMARD), targeted synthetic (tsDMARD), and biological (bDMARD) disease-modifying antirheumatic drugs change the natural course of PsA, allowing the achievement of the therapeutic target in a high proportion of the cases [3].

Remission constitutes a state of inflammation control and lack of symptoms, and it is associated with a lower probability of long-term structural joint damage and consequent functional impairment [3]. There are several scores for PsA in clinical practice that define remission, covering different domains of the disease and quantifying its level of activity [2]. The most commonly used scores are the minimal disease activity (MDA) criteria [4] and the Disease Activity index for Psoriatic Arthritis (DAPSA) [5,6]. MDA requires at least five fulfilled criteria out of a total of seven (regarding the number of painful joints, swollen joints, painful entheses, the extent of skin involvement, visual analogue scale global and pain evaluations as reported by the patient, quality of life). In this system, remission corresponds to the fulfillment of all the seven aforementioned criteria. The MDA criteria are recommended by the T2T International Task Force [7] and the GRAPPA/OMERACT group [8]. Yet, in practice, DAPSA is used more frequently even though it evaluates only the articular peripheral domain. DAPSA includes the number of painful joints, the number of swollen joints, the global and pain VAS patient evaluations, as well as the level of C-reactive protein (CRP) in mg/dL, having a cut-off of 4 for remission and a cut-off of 14 for LDA [6]. Out of the two aforementioned scores, MDA is more stringent when defining remission and LDA [9], being more frequently used in clinical trials, as shown by a recent systematic literature review which analyzed the trials covering this disease published between 2012 and 2019 [10], revealing the use of MDA in 67% of clinical trials and DAPSA in 29.6% of them [9]. These definitions are highly important since the therapy will not be changed because of its ineffectiveness as long as the patients’ disease activity classifies as remission or LDA.

Musculoskeletal ultrasound is a complementary tool to the physical examination [11] when it comes to the screening, diagnosis, monitoring of the disease, as well as during research (EULAR recommendations encourage this aspect [12]), assessing both the inflammatory and the structural changes. Ultrasound is sensitive to treatment effects, but there are few studies that can determine the utility of this type of monitoring. Ultrasound imaging of the joints, entheses, and tendons may bring additional clues in order to consider a patient as being in remission or not. There is no standardized protocol for the ultrasound assessment of joints, tendons, and entheses in PsA (regarding their number and location). There are no validated ultrasound scores to monitor treatment response. The added value of ultrasound for detecting remission is a research question [13]. Power Doppler (PD) is a sensitive ultrasound tool with high reproducibility between evaluators, both in terms of the entheses and the synovial tissue [14], allowing the articular and periarticular inflammation to be visualized. PD appears in the OMERACT-EULAR PDUS score for synovitis, initially validated for rheumatoid arthritis [15] with a quantification from zero to three. Subsequently, the Global EULAR-OMERACT Synovitis Score (GLOESS) [16] was validated in PsA for synovitis. By using this score (GLOESS), the ULTIMATE clinical trial proved its significant decrease in a total of 166 PsA patients after 12 weeks on secukinumab, with a statistically significant differentiation as early as the first week [16,17]. When it comes to the OMERACT definition of enthesitis [18], PD turned out to be important in distinguishing patients with spondyloarthritis, including PsA and other pathologies, compared to grayscale changes (thickening, hypoechogenicity) which could not differentiate between mechanical and inflammatory changes [19]. In the ULTIMATE trial [16], in the first period of the study (0–12 weeks), the enthesitis OMERACT PDUS score had decreased statistically significantly in patients treated with secukinumab under treatment compared to the placebo. In the second period of the study (12–24 weeks), when all patients received active medication, the enthesitis OMERACT PDUS score in the placebo switcher group reached that of the secukinumab patient group. In a study which included psoriasis and PsA patients [20], who were evaluated at baseline and after six months of DMARD treatment, the authors did not detect any PD in any enthesis which was initially evaluated, and no significant improvement regarding the grayscale (GS) appearance of the entheses following treatment. 

Regarding other imaging modalities, there are validated magnetic resonance imaging (MRI) and radiographic scores for monitoring the PsA treatment response. The radiological ones quantify the structural lesions from the axial and peripheral impairment, e.g., the Simplified Psoriatic Arthritis Radiographic Score—SPARS [21]. MRI scores are related to the structural and inflammatory changes in the hands and in calcaneal enthesitis, respectively. Inflammatory and destructive changes in the hands are evaluated by the PsA MRI scoring system (PsAMRIS) [22], while the calcaneus changes are quantified by the heel enthesitis MRI scoring system (HEMRIS) [23]. There is also a validated score of global MRI involvement, namely the Whole-Body Score for Inflammation in Peripheral Joints and Entheses in Inflammatory Arthritis MRI-WIPE [24].

Synovitis and enthesitis can exist subclinically [11], indicating the existence of inflammation which can only be detected by imaging. In this context, the objectives of the study were to evaluate the ultrasound prevalence of enthesitis and active synovitis in a group of PsA patients who reached the therapeutic target under b/tsDMARDs, as well as to evaluate their agreement with a clinical examination of entheses.

## 2. Methods

### 2.1. Patients

In December 2022, the Romanian Rheumatic Diseases Registry (RRBR), which includes all PsA patients in the country (who fulfilled the CASPAR criteria [25]) on b/tsDMARDs, was queried cross-sectionally for PsA patients in DAPSA-defined remission (i.e., DAPSA ≤ 4) and LDA (i.e., DAPSA ≤ 14). Patients needed to live within a maximum distance of 100 km from our rheumatology clinic. All patients fulfilling these criteria were invited to voluntarily participate in the study, which took place between February 2023 and March 2024. All patients offered written informed consent, and the study was approved by the local ethics committee and conducted according to the Declaration of Helsinki.

### 2.2. Evaluations

Each patient underwent on the same day a clinical interview, clinical examination, review of medical history, questionnaires for patient-reported outcomes (0–100 mm visual analog scales for pain and global assessment), peripheral blood sampling, and ultrasound examination. Using data from RRBR, all patients were followed-up after 6 months from baseline clinical and ultrasound assessment for data on relapse (DAPSA > 14) requiring a change in DMARD strategy.

The clinical interview, clinical examination, and review of medical history allowed for the collection of demographic data (sex, date of birth), smoking status, manual labor, weight, height (obesity was defined as a body mass index of 30 kg/m^2^ or above), comorbidities. Arterial hypertension (AHT), type 2 diabetes mellitus (T2DM), hypercholesterolemia, and hyperuricemia were defined by an abnormal blood pressure above 140/90 mmHg, fasting hyperglycemia above 126 mg/dL, total cholesterol or LDL cholesterol above 200/100 mg/dL, and serum uric acid above 7 mg/dL on evaluation or in the presence of specific undergoing pharmacologic treatment, respectively. Regarding psoriasis, diagnosis date, current lesions, and phenotype were recorded. Also, PsA characteristics, such as diagnosis date, history of dactylitis, and type of DMARDs treatment, were noted. The following entheses were examined bilaterally, clinically and by ultrasound: elbow lateral epicondyle, quadriceps insertion, distal patellar tendon insertion, Achilles enthesis, and they were classified as active if the PD signal showed at least a score of 1. Moreover, we examined the joints of hands and feet (radiocubitocarpian—RCC joints, metacarpophalangeal—MCP1–5 joints, proximal interphalangeal—PIP1–5 joints, distal interphalangeal—DIP2-5 joints, and metatarsophalangeal—MTP1–5 joints bilaterally), and the synovitis was classified as active if the PD signal showed at least a score of 1.

In the present study, the aforementioned examination areas were chosen due to good accessibility during ultrasound examination but also in order to facilitate physical examination, which would provide a high degree of reproducibility. 

The ultrasound examination was performed by a single experienced examiner, blinded to clinical examination results, in a dark room, with the Esaote My Lab Twice machine, using a 7–18 MHz linear probe. The evaluation was carried out in GS and PD (with the following settings: a frequency of 750 MHz, a low filter, and the gain below the level that can be detected at the bone level). Active synovitis and active enthesitis were noted according to the OMERACT definitions [18]: active enthesitis was scored when PD grade ≥ 1 plus entheseal thickening and/or hypoechogenicity, while active synovitis was scored when we had PD grade ≥ 1 plus synovial hypertrophy.

### 2.3. Statistics

Data distribution normality was assessed using descriptive statistics, normality, stem-and-leaf plots, and the Lilliefors corrected Kolmogorov–Smirnov tests. Continuous variables are reported as “mean ± standard deviation”, while dichotomous variables are reported as “percentage of sub/group”. The associations between categorical variables were studied using χ^2^ or Fisher’s exact tests. The agreement of clinical and ultrasound examinations was evaluated with: overall agreement, Cohen’s κ (kappa; strength of agreement: κ < 0.2 poor, κ = 0.21–0.40 fair, κ = 0.41–0.60 moderate, κ = 0.61–0.80 good, and κ > 0.80 very good) [26], sensitivity, specificity, and positive likelihood ratio (PLR; effect on increasing probability of involvement detection: PLR > 10 large, PLR = 5–10 moderate, PLR < 5 small) [27]. The statistical tests were considered significant if *p* < 0.05. The statistical analysis was performed using IBM SPSS Statistics version 25.0 for Windows (IBM Corp., Armonk, NY, USA).

## 3. Results

### 3.1. General and Disease Characteristics

The study included 51 patients, women (52.9%), with an average age of 55 years (Table 1). Notably, the sample yielded a high prevalence of obesity (33.3%), smoking (33.3%), AHT (51.0%), and T2DM (17.6%). The PsA patients had established disease with a mean duration of 15 years and a high prevalence of current psoriasis lesions (29.4%) and psoriasis vulgaris phenotype (86.3%). Almost half of the patients were receiving a csDMARD (most frequently methotrexate), three-quarters were on a TNF inhibitor, and more than a third were in DAPSA-defined remission (Table 1).

### 3.2. Clinical and Ultrasound Examination

**Figure 1:**
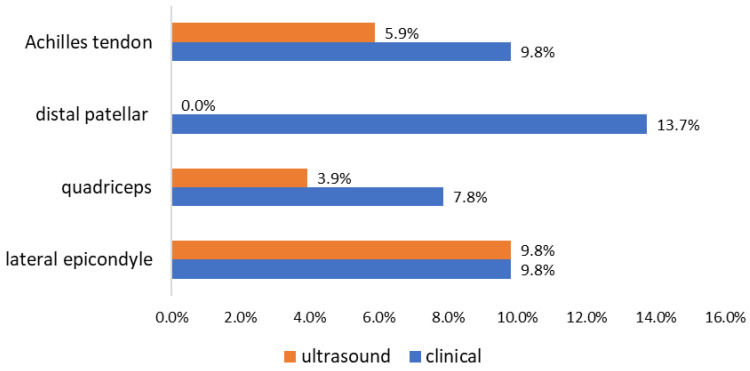
Prevalence of positive clinical and ultrasound findings on enthesis examination (n = 51).

**Figure 2:**
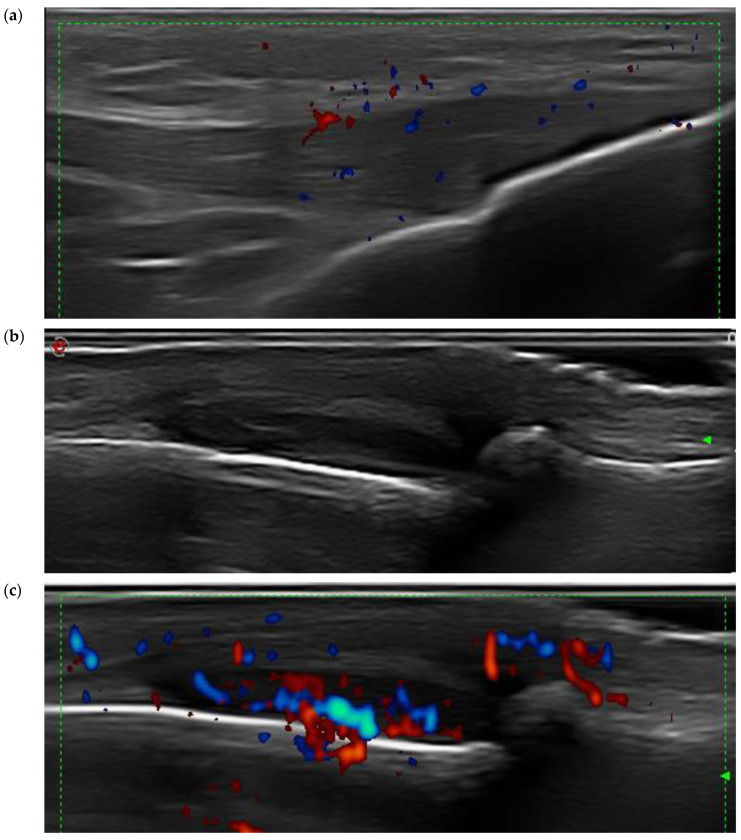
(**a**) PsA patient within treatment target: grayscale (GS) and power Doppler (PD) examination, longitudinal scan of the distal patellar tendon. Abnormal thickening and hypoechogenicity of the enthesis and the presence of PD are noticed. (**b**) PsA patient within treatment target: GS (**b**) and PD examination (**c**), longitudinal scan of the dorsal side of finger 5. Active PD synovitis at the PIP level and active paratenonitis of the extensor of the fifth finger are observed (Esaote MyLab Twice ultrasound device; 18 MHz linear frequency probe).

Although the patients were within the DAPSA treatment target, 11 (21.6%) patients had at least one painful enthesis at clinical examination, 10 (19.6%) patients had ultrasound evidence of active enthesitis (Figure 1 and Figure 2), and 8 (15.7%) patients had ultrasound signs of active synovitis. 

Clinical examination of the right lateral epicondyle proved to have a fair agreement with the ultrasound (Cohen’s kappa of 0.3), with a moderate effect on an increasing probability of involvement detection (PLR = 8.2; Table 2). The clinical examination of the other enthesis sites showed high specificity when compared to ultrasound, but low, absent, or incalculable sensitivity.

**Figure 3:**
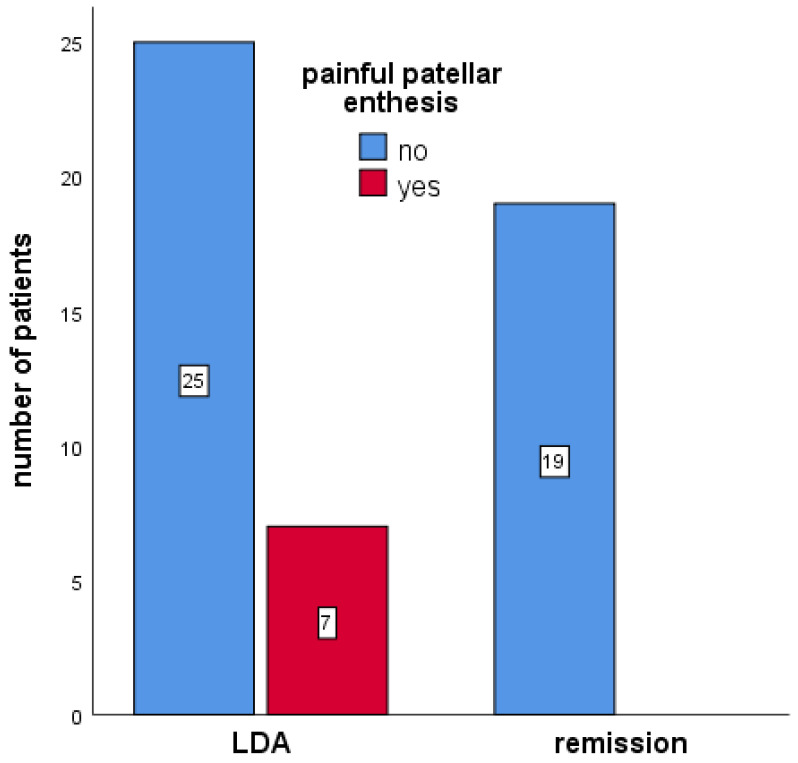
The prevalence of painful patellar enthesis among PsA patients in DAPSA-defined remission or LDA.

Compared to patients in DAPSA-defined remission, patients in LDA had a significantly higher prevalence of painful right patellar enthesitis on clinical examination (21.9% compared to none; *p* = 0.028; Figure 3). There were no other significant differences among patients in remission or LDA regarding clinical examination and ultrasound examination of entheses. 

After 6 months of follow-up, only two patients (3.9%) lost the DAPSA treatment target and needed a change in the DMARD strategy, both of whom had active synovitis at the first ultrasound evaluation. 

Also, if we define US remission as a PD score of zero at the level of the examined joints and enthesis, we can state that 68.5% of patients in DAPSA remission were also in ultrasound remission, and 62.5% of those in DAPSA-LDA were also in ultrasound remission.

## 4. Discussion

Psoriatic arthritis is an aggressive inflammatory condition that may lead to irreversible anatomical damage if not adequately treated. The clinical examination may miss mild but active disease, which can be revealed by imaging such as ultrasound. US is the leading imaging method for screening since it can distinguish synovitis, tenosynovitis, dactylitis, and enthesitis. It is quick, non-invasive, non-ionizing, low cost, and can be reassessed numerous times to complete the clinical examination or to evaluate treatment response [28].

Among all the possible clinical activity indices for PsA, DAPSA was chosen for the present study because it is the only one reported in the RRBR database. An advantage of DAPSA is the fact that it has been developed for easy and quick execution (under 10 min), assessing peripheral articular involvement of PsA, with the disadvantage of not counting for skin and enthesis involvement [2].

By limiting the analysis to patients with a DAPSA-defined therapeutic target (remission and LDA), the study aspired to fill a gap in the literature, since a recent systematic literature review of ultrasound changes in PsA patients during remission identified only seven articles [29].

A recent multi-center international trial showed that among the elements of an enthesitis, defined according to OMERACT, the presence of a PD signal in entheses in spondyloarthritis showed good reliability [30]. This is the reason why in our study, we used PD to define active enthesitis and synovitis.

Relatively similar data to those of the present study were published by Ruta et al. with a rate in patients with PsA in remission of 30% US synovitis with PD grade ≥ 1 in at least 1 joint, but it must be mentioned that the authors scanned 18 joints per patient [31], while we scanned 40 joints per patient.

In the literature there are published data in which most PsA patients in remission one year after initiating an anti-TNF agent presented a positive signal when using contrast-enhanced ultrasound [32], indicating the persistence of synovial inflammation. The PD ultrasound remission was associated with DAS28-ESR remission, but not with Boolean or DAPSA remission [33].

Ultrasound is superior to physical examination thanks to its ability to detect subclinical enthesitis. Michelsen et al. [34], while studying the Achilles tendon in PsA patients, found a prevalence of subclinical enthesitis of 16%. In the same study, no association was found between clinical enthesitis and the ultrasound elements of enthesitis. Elements of subclinical synovitis were also detected in patients in remission, defined according to DAS28 or MDA [35]. Freeston et al. [36] analyzed patients with early PsA and reported enthesis PD in just 5.1% of cases, most frequently in the Achilles and the lower patellar tendon. In total, 17% of entheses were painful, while 4% of the clinically non-painful entheses showed activity (GS > 1 and/or PD > 0). All subclinical enthesitis were detected in the lower limbs. The physical examination overestimated activity in 28 out of 214 entheses (14%).

Large discrepancies were reported between ultrasound and clinical scores in a high proportion of PsA patients in remission, who showed activity on ultrasound. The Boolean remission and DAPSA were the criteria used for defining clinical remission, and regarding the ultrasound examination, minimal ultrasound disease activity, defined as a PD score of up to one in joints, entheses, or tendons, was used [35]. The discrepancy between physical examination and ultrasound changes was also highlighted by other trials which either focused only on the Achilles tendon [34] or which took into consideration several entheses and joint locations with quantification of the clinical (MDA, DAPSA, and Boolean’s remission criteria) and ultrasound remission indices [37]. Even regarding the clinical remission criteria, a phase 3 trial with tofacitinib has identified at month 6, a moderate agreement between DASPA-defined remission and VLDA and between DAPSA-defined LDA and MDA, respectively [38].

It is known that clinical indices are not specific as they can be positive in fibromyalgia or in the case of mechanical impairment [39]. In patients with PsA and fibromyalgia, higher clinical disease scores are registered [40,41]. A number of fibromyalgia points overlap with enthesitis points. Fiorenza et al. clinically used the Maastricht Ankylosing Spondylitis Enthesitis Score (MASES) and the Leeds Enthesitis Index (LEI). A high percentage of clinical enthesis pain and ultrasound anomalies were also found and quantified by the GUESS score. High clinical scores were reported in 43% of PsA patients and in 50.8% of fibromyalgia patients, respectively, while ultrasound anomalies were reported in 77% and 35%, respectively, but erosions and PD ultrasound managed to distinguish between PsA and fibromyalgia patients.

Following neoangiogenesis triggered by inflammation through prostaglandin E2, neurogenesis also occurs in the enthesis, leading to pain, which cannot be assessed by imaging. Even if inflammation is controlled and the structural changes are remitted, the pain generated by neurogenesis may persist [42], explaining the present discrepancy in most trials between the clinical evaluation (pain being a part of the clinical indices) and imaging [7].

The fact that the current study observed more active enthesitis compared to synovitis was consistent with the result of a recent study that showed that PD in enthesitis was less responsive compared to PD in synovitis following 3 and 6 months since initiating bDMARDs in a group of PsA patients [43,44,45], suggesting that enthesitis is a difficult-to-treat domain of PsA.

Regarding the prediction of ultrasound data, Polacheck et al. proved in a cross-sectional trial the correlation between MASEI ultrasound score and the radiological scores of axial and peripheral PsA involvement [46]. Another prospective study with a duration of 12 months highlighted that the radiological progression in entheses in a group of 83 PsA patients was influenced both by the duration of the disease and by the ultrasound presence of enthesophytes at baseline, without proving a correlation with a different element which falls within the definition of an enthesitis [47]. In these trials, patients were not divided according to disease activity levels. In a group of 54 PsA patients in remission, the presence of active synovitis on ultrasound could also predict a flare episode [31].

There are currently no data supporting the escalation of therapy based on the ultrasound of the upper limb in PsA patients in remission, but negative ultrasound results may serve as an indicator to taper treatment [29].

## 5. Conclusions

In patients in remission, musculoskeletal ultrasound timing must be decided in order to be applied during the therapeutic management process. A high proportion of ultrasound-active enthesitis and synovitis was detected in PsA patients considered within the DAPSA-defined therapeutic target. Compared to the higher standard (ultrasound), the performance of the clinical examination was very low for the detection of enthesitis (the lateral epicondyle had the best performance: Cohen’s kappa of 0.3 and PLR of 8.2). The discrepancy between DAPSA and ultrasound changes in active synovitis and enthesitis in PsA patients could be followed for possible relapses and could be the basis for a standardized protocol for using ultrasound to monitor PSA progression.

## Figures and Tables

**Table 1:** Sample characteristics (n = 51).

**General (n = 51)**	
Women (n, %)	27 (52.9%)
Age at evaluation (years)	55.0 (14.7)
BMI (kg/m^2^)	27.5 (4.9)
Obesity ^&^ (n, %)	17 (33.3%)
Smoking (n, %)	17 (33.3%)
Manual labor (n, %)	8 (15.7%)
AHT * (n, %)	26 (51.0%)
T2DM * (n, %)	9 (17.6%)
Hypercholesterolemia * (n, %)	18 (35.3%)
Hyperuricemia * (n, %)	6 (11.8%)
**PsA (n = 51)**	
Duration (years)	15.0 (8.7)
Dactylitis (n, %)	15 (29.4%)
csDMARDs (n, %)	49.0%
- Methotrexate (n, %)	20 (80.0%)
- Leflunomide (n, %)	5 (20.0%)
b/tsDMARDs (n, %)	51 (100%)
- Anti-TNFα (n, %)	38 (74.5%)
- Anti-IL-17 (n, %)	9 (17.7%)
- Anti-IL-23 (n, %)	2 (3.9%)
- JAKi (n, %)	2 (3.9%)
DAPSA	6.2 (4.5)
- Remission ** (n, %)	19 (37.3%)
- LDA ** (n, %)	32 (62.7%)
Target ** duration (years)	3.7 (2.7)
**Psoriasis (n = 51) ^#^**	
Duration (years)	21.9 (12.7)
Current lesions (n, %)	15 (29.4%)
Vulgaris (n, %)	44 (86.3%)
Palmoplantar (n, %)	5 (9.8%)
Scalp (n, %)	40 (78.4%)
Nail (n, %)	25 (49.0%)

^&^ Obesity is defined as BMI > 30 kg/m^2^; * AHT, T2DM, hypercholesterolemia, and hyperuricemia are defined, respectively, by abnormal blood pressure (>140/90 mmHg), fasting hyperglycemia (>126 mg/dL), total cholesterol or LDL cholesterol above 200/100 mg/dL, serum uric acid > 7 mg/dL on evaluation or in the presence of specific current pharmacologic treatment. ^#^ There were 2 patients (3.9%) diagnosed with PsA without psoriasis. ** Remission (DASPA ≤ 4) and LDA (4 < DAPSA ≤ 14) represent the target; Continuous variables are reported as “mean (SD)”. Categorial variable are report as fraction from group (n = 51) or subgroup (designated by “-”); Abbreviations: AHT—arterial hypertension; BMI—body mass index; b/tsDMARDs—biological or targeted synthetic disease-modifying antirheumatic drugs; JAKi—Janus kinase inhibitors; IL—interleukin; LDA—low disease activity; LDL—low-density lipoproteins; PsA—psoriatic arthritis; SD—standard deviation; T2DM—type 2 diabetes mellitus; TNF—tumor necrosis factor.

**Table 2:** Concordance of clinical and ultrasound evaluations.

	Se	Sp	OA	PLR	Ck	p (Ck)
rLE	50.0%	93.9%	92.2%	8.2	0.297	0.024
lLE	0	93.8%	90.0%	0	0.062	0.655
rCV	0	94.0%	92.2%	0	0.030	0.801
lCV	0	96.0%	94.1%	0	0.027	0.838
rP	-	86.3%	86.3%	-	-	-
lP	-	94.1%	94.1%	-	-	-
rA	-	92.2%	92.2%	-	-	-
lA	0	97.9%	92.2%	0	0.030	0.801

Note: *p* values represent the significance level of calculated Ck indices. Abbreviations: r/lA—right/left Achilles; Ck—Cohen’s kappa; r/lCV—right/left quadriceps; r/lLE—right/left lateral epicondyle; OA—overall agreement; r/lP—right/left patella; PLR—positive likelihood ratio; Se—sensitivity; Sp—specificity.

## Data Availability

The data presented in this study are available on request from the corresponding author due to patient confidentiality.
